# The General Medical Council

**Published:** 1897-12-04

**Authors:** 


### THE GENERAL MEDICAL COUNCIL.

The General Medical Council commenced its sixty-third session on Tuesday, November 23rd, and did so, for the first time for 34 years, in the absence of Sir Richard Quain, the president. The first business was to move that Sir William Turner should take the chair, in order that the new members, Sir Christopher Nixon and Mr. Victor Horsley, might be formally admitted and the Council fully constituted; which having been done, a letter from the absent president was read, and, in accordance with a suggestion which it contained, Sir William Turner was appointed chairman for the session. The letter contained the president's usual summary of the expected business, and it was ordered to be entered upon the minutes, together with an expression of sympathy with the president in his illness, and of regret at his unavoidable absence from the chair. The ordinary tables showing the results of competitive examinations for medical appointments in the public services were then received and acknowledged; and, as the Director-General of the Army Medical Department had expressed his regret that he was unable to accede to a request made by the Council (namely, that he would notify where the preliminary examination in arts had been passed, and the date of such examination, in the case of any candidate who evinced marked deficiency in preliminary education on the occasion of his examination for admission into the Army Medical Department) inasmuch as, to his mind, there were serious objections to dealing with this subject in the manner proposed, Dr. Heron Watson moved, and Sir Richard Thorne-Thorne seconded, that the letter of refusal should be referred to the Examination Committee for consideration, and for report during the session. This having been assented to, the Council proceeded to consider the question of the employment, by medical men, of unqualified assistants to attend the sick, and discussed a resolution, which was carried on the following day, and which we have commented upon elsewhere, declaring the practice to be essentially fraudulent, and that anyone convicted of it would be liable to removal from the Medical Register for conduct adjudged to be "infamous in a professional respect." The debate was adjourned at four o'clock, in order to allow of the assembling of committees.

On Wednesday the adjourned debate was resumed and concluded, and the Council, having accepted the conclusion of its Executive Committee that it was not at present necessary to pass a resolution limiting the duration of speeches, was informed by its Business Committee that a petition had been sent in, professing to emanate from "Registered Practitioners of Great Britain and Ireland," but signed by only 53 persons, which did not conform to the standing orders of the Council in respect of petitions, and which therefore could not be received. The order is that no petition can be received which deals with more than a "single main question," whereas the one under consideration dealt with "ten or more," and it further appeared that there would be an application from a deputation to present it and to speak in its support. Mr. George Brown moved, and Sir W. Thomson seconded, that the standing order should be suspended and the petition received, but this was not carried, and the matter dropped. The next business was a report from the Executive Committee on applications for restoration to the Register by persons who had been removed from it. In reference to one of these, that of Mr. Theobald, the Council ordered him to be informed that, as he no longer possessed a registrable qualification, there was no power to comply with his petition; and another case stood over for reference to the legal advisers of the Council. A letter was read from the Privy Council, saying that there was "no necessity" for the Council to consider the provisions of the Midwives Registration Bill of the last session of Parliament; but the Council decided to keep alive its committee on this question, to be in readiness to receive and report upon any future Bill for the same purpose which may be submitted to it by Government. A general resolution, proposed by Dr. McVail, and seconded by Dr. Atthill, declaring that the practice of midwifery cannot be safely entrusted to persons possessing a less knowledge of midwifery and of medicine and surgery than is required for admission to the Medical Register, was not accepted by the Council; and, after receiving a report from a committee appointed to urge upon Government that fines under the Medical Acts, levied within the boundaries of the metropolis and of some other places, should be paid to the Council instead of, as at present, to the police, the meeting adjourned.

On Thursday a good deal of time was occupied in considering, *in camera*, a recommendation from the Penal Cases Committee; and then the Council heard a complaint of covering an unqualified person, brought by the Medical Defence Union and the Wigan Medical Guild, against George Hamilton Wyse, of Southport. The complaint was declared to be proved, but the Council adjourned its further consideration until next session, without resolving that the conduct complained of was "infamous." The effect of this adjournment will be to give the defendant an opportunity of proving to the Council that he has abandoned the practices complained of.

On Friday penal cases were proceeded with. William John Patterson was removed from the Register in consequence of having been convicted of an indecent assault, for which he had been sentenced to nine months' imprisonment, with hard labour; and William Robert Nuttall Maloney was removed for improper advertising, in association with an electrical quack. Charges of covering unqualified persons were proved against Charles Day and against William Masson, but both these cases were dealt with by adjournment, as in that of Wyse above-mentioned. A similar decision was arrived at on Saturday, as the result of the investigation of a charge of covering brought against Julius Mostertz by the Medical Defence Union on Monday.

On Monday Dr. J. B. Tuke moved the adoption of the report of the Education Committee on measures to secure a higher standard of preliminary examination. The following recommendations were adopted: (1) "That the Council intimate to all the examining bodies whose examinations in general education are recognised by the Medical Council, as qualifying for the registration of medical students, that, on and after the first day of January, 1899, no certificate of lower standard than that of the matriculation or entrance and the junior local examinations of the Universities of the United Kingdom will be accepted for registration." (2) "That the Education Committee be instructed to revise the list of examinations recognised by the Council, with a view to removing from it on the first day of January, 1899, those examinations which are of a lower standard than that indicated in recommendation 1." It was further decided to omit logic from the list of optional subjects after January 1st, 1899.

After the reception of a report from the Executive Committee on the penal or disciplinary powers of the qualifying medical authorities, the Council proceeded to consider certain penal business, and then adjourned.

